# Reliability of Electrical Threshold Testing for Assessing Sensory, Motor and Pain Thresholds: An Exploratory Study in Active Subjects

**DOI:** 10.3390/jfmk11030267

**Published:** 2026-07-07

**Authors:** Izarbe Ríos-Asín, Elena Bueno-Gracia, Isabel Albarova-Corral, Pilar Pardos-Aguilella, Gianluca Ciuffreda, Miguel Malo-Urriés

**Affiliations:** PhysiUZerapy Health Sciences Research Group, Department of Physiatry and Nursing, Health Sciences Faculty, University of Zaragoza, 50009 Zaragoza, Spain; irios@unizar.es (I.R.-A.); ebueno@unizar.es (E.B.-G.); ialbarova@unizar.es (I.A.-C.); ppardos@unizar.es (P.P.-A.);

**Keywords:** electrical threshold testing, QST, reliability, electrical stimulation, sensorimotor function, sports-related injuries

## Abstract

**Background**: Sensorimotor impairments are common following sports injuries. Electrical Threshold Testing (ETT) is a promising quantitative sensory testing (QST) tool that allows the assessment of sensory deficits, motor recruitment, and pain perception. Although various protocols have been proposed, direct comparisons between studies and protocols remain difficult due to methodological inconsistencies, particularly regarding the number of measurements used, which significantly affect reliability. The main objective was to determine the number of trials required to obtain reliable measurements of electrical sensory threshold (EST), electrical motor threshold (EMT), and electrical pain threshold (EPT) in terms of both intra- and interday reliability. **Methods**: In this repeated-measures study, 14 active participants underwent three electrical stimulation protocols (sensory, motor, and pain) and performed five measurements per threshold. Averages of 1 to 5 measurements were analyzed using intraclass correlation coefficients (ICCs). **Results**: For EST, averaging multiple trials yielded good-to-excellent intra- and interday reliability (ICCs = 0.778–0.964). For EMT, intraday reliability was excellent (ICCs > 0.928), but interday stability remained moderate depending on the performed test. For EPT, intraday reliability was good to excellent (ICCs = 0.812–0.957), whereas interday stability (ICCs > 0.782) required averaging at least three trials. Single-trial assessments provided insufficient precision across all thresholds. **Conclusions**: Implementing a standardized protocol of three-averaged trials, including EST, EMT and EPT, appears to be an optimal, reliable, and time-efficient balance for clinical settings. These findings may contribute to ETT standardization in active populations, enhancing clinical efficiency, and supporting its use as a reliable tool for assessing sensorimotor impairments and monitoring treatment outcomes.

## 1. Introduction

Sensorimotor system alterations, defined as disruptions in the integration and processing of sensory input and motor responses within the peripheral and central nervous system [1], have been frequently reported following sport-related injuries. Traumatic events following contact sports, such as repetitive neurotrauma, may exert a pathophysiological impact on sensorimotor perception and motor control [2]. Likewise, overuse conditions, including tendinopathies, highlight the prominent role of peripheral and central sensitization in the pain symptoms experienced by athletes [3]. These impairments include altered sensitivity, deficits in muscle strength and performance due to reduced motor unit recruitment, and dysfunction of the endogenous pain modulation system [3]. Such alterations may compromise rehabilitation outcomes and negatively influence subsequent physical capacity.

Quantitative Sensory Testing (QST) is a method previously used to assess sensorimotor function of both the peripheral and central nervous system in athletes and the active population [4,5]. Among its modalities, electrical threshold testing (ETT) has emerged as a complementary tool to mechanical or thermal QST [6]. Electrical thresholds include electrical sensory threshold (EST)—defined as the minimum current intensity needed to induce conscious perception of electrical stimulation—electrical motor threshold (EMT)—referred to as the current intensity required to elicit a visible motor response in the muscles—and electrical pain threshold (EPT)—defined as the minimum intensity of current that induces the first perceptible pain sensation. ETT offers several key advantages, such as greater objectivity, improved reproducibility, and reduced examiner-dependent variability [7]. Furthermore, this approach is less influenced by environmental factors and is easier to standardize, as it does not require the use of specifically calibrated mechanical or thermal devices [8]. In addition, ETT allows the assessment of recruitment patterns across different nerve fiber types, facilitating the identification of sensory, motor, or pain-related impairments [9]. This capability may enhance the diagnostic process of sensorimotor dysfunction and assist clinicians in selecting more targeted therapeutic strategies to optimize rehabilitation and promote recovery following sport-related injuries.

Despite its advantages, ETT still lacks a standardized protocol when used for active populations. Variability in its application arises from differences in electrode type, stimulation site, electrical parameters, and methodological constraints, such as the repetitions required for a reliable measurement [10]. Several approaches have been described in the literature, with distinct procedures aimed at improving accuracy and reproducibility. Some protocols involve repeating the ETT until a consistent response is observed within a narrow range (e.g., ±2 μA), ensuring stability of the sensory thresholds [11,12]. Others apply the method of limits, where electrical stimulation is applied with incremental changes in intensity, and participants indicate when the stimulus is first perceived or no longer felt during alternating ascending and descending sequences. The final threshold value is calculated as the average of multiple trials (e.g., 6 trials) [7]. Another widely used method involves increasing current intensity until the required sensation or motor recruitment is elicited, then removing current and repeating this process two to three times. In some cases, the lowest intensity of two [8] or three [13] trials is recorded. Alternatively, the mean of two [14] or three [6,9] trials is used for further analysis.

These varied methodologies reflect ongoing efforts to standardize ETT. However, the considerable methodological variability hinders the establishment of consistent clinical guidelines in active populations, as different protocols—although demonstrating acceptable interrater and intrarater reliability [15]—are not uniformly applied. This inconsistency in the literature also impedes the establishment of normative reference values, which could be valuable in sports medicine for diagnosing sensorimotor disorders following sports-related injuries and for monitoring their progression during return to sport [16]. Furthermore, such variability compromises the comparability of findings across studies, ultimately limiting the overall consistency and practical applicability of ETT.

Therefore, it is essential to define the methodological parameters required for the assessment of electrical thresholds in active populations, providing clear and practical recommendations that are adaptable to commonly used clinical devices, as well as to the time and information constraints associated with the evaluation of sport-related sensorimotor conditions. In this context, the number of measurements required remains inconsistent across protocols, highlighting the need for standardization to optimize time efficiency, ensure reliable outcomes, and facilitate the generation of comparable data on a global scale. The main objective of this pilot study was to determine the number of trials required to obtain reliable measurements of EST, EMT, and EPT both within the same day (intraday reliability) and across two consecutive days (interday reliability) in active subjects.

## 2. Materials and Methods

### 2.1. Study Design

An observational, descriptive, cross-sectional, prospective pilot study was designed. The study was approved by the Research Ethics Committee of the Autonomous Community of Aragón (CEICA) (C.I. PI24/248; approval date: 29 May 2024) and received authorization for the processing of personal study data from the Data Protection Unit of the University of Zaragoza (CUSTOS), with reference number RAT 2024-142. All participants signed an informed consent form in accordance with the Declaration of Helsinki [17].

### 2.2. Participants

A convenience sample of 14 healthy adult volunteers was recruited. To be eligible for the study, participants were required to be aged 18 years or older, to have the ability to communicate and to understand the tests performed and to provide written informed consent. Participants were required to be in self-declared good health, with no underlying medical conditions that could potentially influence the study outcomes, and to be physically active according to the World Health Organization, engaging in at least 150 min of regular moderate-intensity exercise or sports activities per week [18]. Participants were excluded if they had a history of chronic disorders, including endocrine, neurological, psychiatric, urogenital, or musculoskeletal conditions, or any other condition that could interfere with results.

### 2.3. Electrical Threshold Testing

Three assessment sessions were scheduled across the study design. In the first session, which was considered the baseline, participants completed a questionnaire to collect demographic data. The second session was conducted on the same day, one hour after the first session, to ensure adequate physiological recovery and minimize potential carryover effects. Finally, the third session was performed on the following day, exactly 24 h after the initial assessment.

The ETT was performed in the three sessions to assess reliability. The main outcomes were the EST, defined as the intensity (mA) at which the participant first perceived the electrical stimulation; EMT, the intensity (mA) at which a visible motor response was induced; and EPT, the intensity (mA) required to elicit the first pain perception in the participant. Thresholds were measured using a low-frequency symmetrical biphasic current with disposable, square, 25 cm^2^ electrodes, a frequency of 100 Hz and a pulse width of 100 µs. The intensity was increased at a rate of 1 mA/s across all performed tests. A GYMNA MYO 200 electrotherapy device (GymnaUniphy, Bilzen, Belgium) was used for assessment.

Throughout the procedure, participants remained in a supine position, with their dominant forearm in supination [9]. Measurements were conducted on the dominant forearm, chosen as a representative area for evaluating sensory perception. The electrodes were positioned on the anterior side, following the wrist flexor muscle’s longitudinal alignment. Specifically, the distal electrode was placed 4 cm above the wrist joint line, while the proximal one was located 4 cm below the elbow fold [19]. To reduce variability between examiners and maintain procedural consistency, the same primary researcher was responsible for placing and removing all electrodes.

### 2.4. Assessment Procedure

Three progressive stimulation tests were performed in each assessment session:

- Sensory Threshold Test (STT): Electrical stimulation was increased until the EST was reached. Five consecutive measurements of EST were taken with a neural recovery time of 0 s, as in a previous pilot study [20].
- Motor Threshold Test (MTT): Electrical stimulation was increased until a visible motor response in the flexor digitorum superficialis muscle was elicited (EMT). EST was registered when the participant first reported the electrical stimulation, and EMT was recorded when the muscle response was reached. Five consecutive measurements were taken with a recovery time of 30 s, as in a previous study [20].
- Pain Threshold Test (PTT): Electrical stimulation was increased until the participant reported the first pain sensation (EPT). EST, EMT and EPT were recorded. Five consecutive measurements were taken with a recovery time of 90 s, as in a previous study [20].

All measurements were performed by the same experienced examiner in a controlled environment to avoid risk of bias.

### 2.5. Statistical Analysis

Statistical analysis was conducted using IBM SPSS Statistics (version 29.0, IBM Corp., Armonk, NY, USA). Descriptive analyses were performed to characterize the demographic variables of the sample. For quantitative variables, the mean and standard deviation were reported, whereas for categorical variables, absolute frequencies and percentages were calculated.

For each test (STT, MTT, PTT), the mean values derived from 1 to 5 repeated measurements were used to calculate reliability values. To assess intraday reliability, measurements from session 1 (baseline) and session 2 were compared, whereas interday reliability was determined by comparing session 1 (baseline) and session 3. Intraclass Correlation Coefficients (ICCs) along with their 95% confidence intervals were computed using a two-way mixed model and absolute agreement. The interpretation of ICC values followed established criteria: >0.90 = excellent, 0.75–0.90 = good, 0.50–0.75 = moderate, and <0.50 = poor reliability [21]. Additionally, the Standard Error of Measurement (SEM) and the Minimal Detectable Change (MDC) quantified the absolute measurement error and clinical stability. The SEM was calculated using the formula $SEM=SD_{baseline}\times \sqrt{1-ICC}$, where *SD_baseline_* represents the standard deviation of the initial measurement. The MDC, reflecting the minimum change required to detect a true clinical difference at a 95% confidence level, was calculated as $MDC=SEM\times 1.96\times \sqrt{2}$. A *p*-value < 0.05 was considered indicative of statistically significant differences.

## 3. Results

### 3.1. Descriptive Analysis of the Sample

The sample consisted of 14 participants. Table 1 presents the analysis of the descriptive variables. The mean age of the sample was 23.57 ± 4.18 years, with an average height of 1.63 ± 0.12 m and an average body mass of 69.14 ± 27.64 kg. Of the participants, 71.40% were female, and 100% were right handed.

### 3.2. Sensory Threshold Test

**Figure 1:**
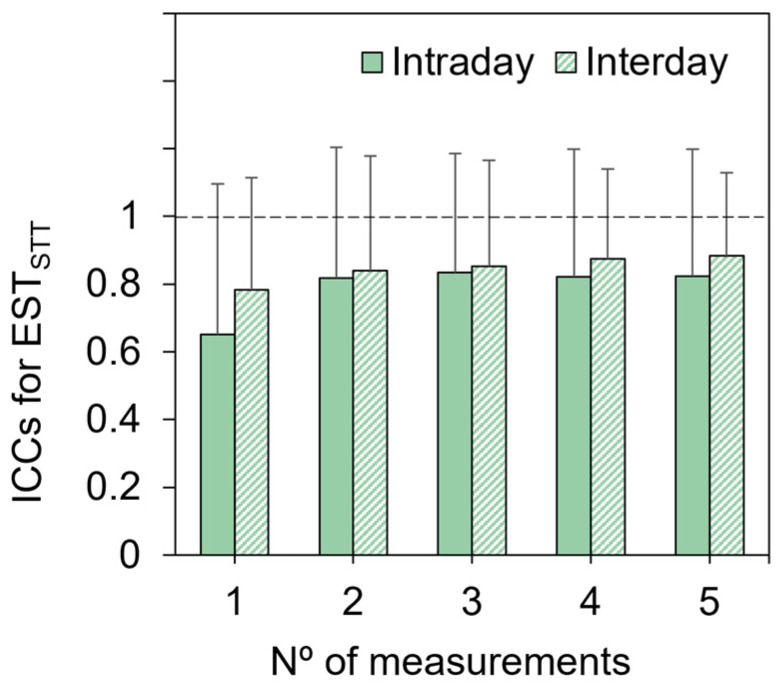
Intra- and interday reliability analysis of the measures for sensory threshold yest. 1–5 refer to the number of measures used to obtain the mean. The dashed line reflects the upper bound of the Intraclass Correlation Coefficient (ICC = 1). EST: Electrical Sensory Threshold; ICCs: Intraclass Correlation Coefficients; STT: Sensory Threshold Test.

Table 2 represents the reliability values for the STT, including ICC, SEM and MDC. Reliability analysis of the STT demonstrated moderate and good intraday and interday ICCs when using the mean of a single measurement (0.651 and 0.783, respectively). When the mean was calculated using multiple measurements, the ICC values increased (0.817–0.884), while SEM and MDC values decreased (0.60–0.74 mA and 1.66–2.05 mA, respectively). The greatest intraday reliability was observed with the average of three measurements (ICC = 0.834; SEM = 0.66 mA; MDC = 1.84 mA), while the greatest interday reliability was achieved using the average of five measurements (ICC = 0.884; SEM = 0.60 mA; MDC = 1.66 mA). Figure 1 represents the ICCs intra- and interday for STT.

### 3.3. Motor Threshold Test

**Figure 2:**
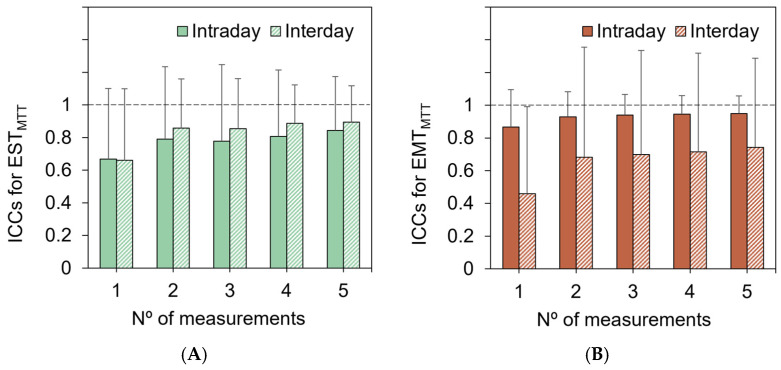
Intra- and interday reliability analysis of the measures for motor threshold test. (**A**): reliability for electrical sensory threshold; (**B**): reliability for electrical motor threshold. 1–5 refers to the number of measures used to obtain the mean. The dashed line reflects the upper bound of the Intraclass Correlation Coefficient (ICC = 1). EMT: Electrical Motor Threshold; EST: Electrical Sensory Threshold; ICC: Intraclass Correlation Coefficient; MTT: Motor Threshold Test.

Table 3 shows the reliability analysis for MTT. Reliability indexes of the second test showed moderate-to-excellent intraday reliability, with the greatest values observed when averaging five measurements for both EST (ICC = 0.844; SEM = 0.94 mA; MDC = 2.59 mA) and EMT (ICC = 0.949; SEM = 0.75 mA; MDC = 2.09 mA). Regarding interday reliability, good ICC values were found for EST when the mean was calculated using multiple measurements (ICC = 0.855–0.895), while EMT showed moderate reliability under the same conditions (ICC = 0.683–0.742). When only a single measurement was used to calculate the mean, interday reliability was moderate for EST (ICC = 0.660; SEM = 1.36 mA; MDC = 3.76 mA) and poor for EMT (ICC = 0.460; SEM = 2.56 mA; MDC = 7.08 mA). Figure 2 represents the ICCs intra- and interday for MTT.

### 3.4. Pain Threshold Test

**Figure 3:**
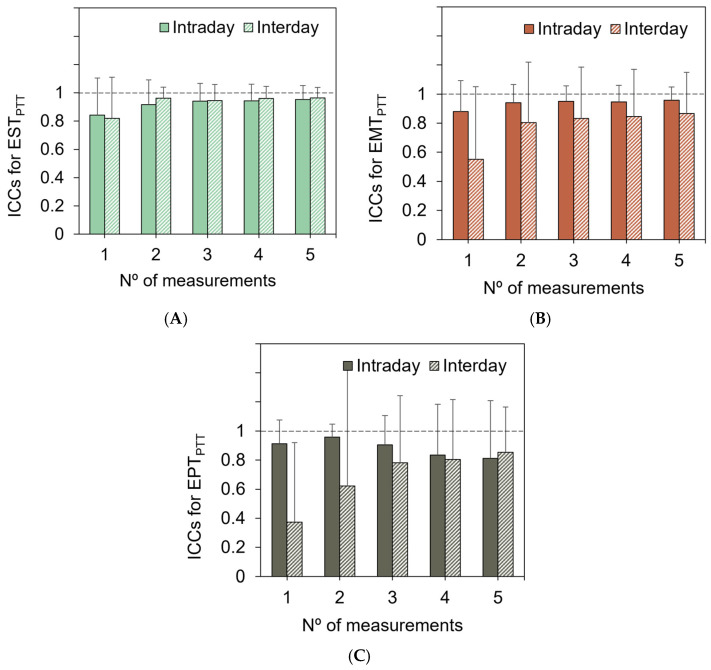
Intra- and interday reliability analysis of the measures for pain threshold test. (**A**): reliability for electrical sensory threshold; (**B**): reliability for electrical motor threshold; (**C**): reliability for electrical pain threshold. 1–5 refer to the number of measures used to obtain the mean. The dashed line reflects the upper bound of the Intraclass Correlation Coefficient (ICC = 1). EMT: Electrical Motor Threshold; EPT: Electrical Pain Threshold; EST: Electrical Sensory Threshold; ICC: Intraclass Correlation Coefficient; PTT: Pain Threshold Test.

Table 4 shows the reliability results for PTT. The reliability analysis of the PTT demonstrated good-to-excellent intraday reliability for all thresholds. The greatest values were observed when averaging five measurements for EST (ICC = 0.952; SEM = 0.49 mA; MDC = 1.37 mA) and EMT (ICC = 0.958; SEM = 0.54 mA; MDC = 1.49 mA), and two measurements for EPT (ICC = 0.957; SEM = 0.60 mA; MDC = 1.67 mA). For interday reliability, good-to-excellent ICC values (ranging from 0.803 to 0.964) were observed for most EST and EMT outcomes, except for EMT when only a single measurement was used (ICC = 0.552). The analysis comparing interday EPT revealed good reliability (ICCs ranging from 0.782 to 0.853) when the mean was calculated with three or more measurements. Figure 3 represents the ICCs intra- and interday for PTT.

## 4. Discussion

Despite the potential use of ETT as a QST tool for somatosensory impairments, methodological heterogeneity remains evident across existing protocols, particularly regarding the number of measurements used to determine EST, EMT or EPT. This variability limits the comparability of findings across studies and hinders the development of standardized clinical recommendations for active populations. Therefore, identifying the minimum number of measurements required to achieve reliable outcomes is clinically relevant, as it may improve testing efficiency while ensuring reliable measurements.

The findings of this exploratory study revealed distinct reliability profiles across the evaluated electrical thresholds, demonstrating that the stability of the measurements depends on both the specific threshold type and the number of repetitions averaged. For the EST, preliminary data suggest that a mean of at least two measurements may be sufficient to obtain good reliability across all tests performed (ICC = 0.778–0.964). For the EMT, good-to-excellent reliability was observed when averaging multiple trials, except for interday reliability in MTT. Within this test, EMT remained moderate across all averaging conditions, approaching but not exceeding the baseline criterion for good reliability [21] even when pooling five attempts (maximum ICC = 0.742). For the EPT, an average of 1–3 measurements provided excellent intraday stability (ICC range = 0.905–0.957), whereas establishing good interday reliability required a higher number of repetitions (3–5; ICC range = 0.782–0.853). Taken together, these preliminary patterns suggest that a standardized three-measurement protocol might represent a balanced option to optimize overall reliability across thresholds and feasibility in clinical practice. However, it should be interpreted cautiously given the exploratory scope.

The preliminary results for the EST demonstrated good-to-excellent intra- and interday stability (ICCs = 0.778–0.964) when averaging multiple measurements. Crucially, data indicated that relying on a single trial appeared insufficient to achieve acceptable precision for EST in active subjects, aligning with previous observations where isolated measurements yielded poor reproducibility [22]. While some literature using specialized pin electrodes reported lower interday reliability for sensory thresholds [7], the present standardized protocol, together with previous literature, suggested that when using conventional surface electrodes, averaging at least two [8] or three [13,23,24] measurements might offer a highly reliable baseline for sensory assessment. Interestingly, the EST exhibited higher reliability values when evaluated within the PTT (ICCs = 0.916–0.964 with multiple measurements). This outcome may be attributed to experiencing a wider range of electrical stimulation intensity in PTT, which may allow subjects to more easily differentiate the initial sensory onset from discomfort or motor recruitment, thereby reducing intra-individual criteria shifting.

Regarding the EMT, it showed good-to-excellent reliability in PTT when averaging multiple measurements, while its interday reliability remained moderate in MTT for multiple trials. This lower interday stability aligned with previous literature reporting greater coefficients of variation across different days compared to single-day assessments [25]. From a methodological perspective, this discrepancy may be attributed to the challenges of standardizing the visual detection of the initial motor contraction between sessions. While an examiner can easily maintain a consistent visual baseline on the same day, changes in electrode repositioning or skin impedance between days can alter the recruitment of motor units, leading to slight variations in the observed motor response [26,27]. Additionally, EMT reliability values seemed to improve when the threshold was evaluated within the PTT rather than the MTT. Because the electrical intensity required to reach EPT far surpasses the initial motor onset, it induces a complete motor response [27]. This recruitment might facilitate the identification of a clearer visual landmark of the flexor digitorum superficialis muscle, minimizing examiner subjectivity across sessions. Although a systematic review supported the potential reliability of clinically observed EMT [28], its interday moderate stability demands caution, and future research should focus on optimizing visual standardization and incorporating objective physical markers to enhance intersession precision.

EPT reliability demonstrated good-to-excellent intraday values (ICC = 0.812–0.957) in all cases and good interday values (ICC > 0.782) when averaging three or more measurements, aligning with previous literature that supports the reliability of three-trial pain assessments [23,24]. The observed discrepancy between intra- and interday reliability may be attributed to the multifactorial nature of pain perception, which is influenced by a variety of factors that fluctuate across different days, such as autonomic and hormonal activity or psychological state [29,30]. Another interesting finding was the gradual reduction in intraday reliability (from excellent to good) as the number of measurements increased. This decline may be related to the modulatory effects of low-frequency biphasic electrical stimulation on pain pathways, which may induce both primary and secondary analgesia, as well as influence peripheral and central sensitization [31]. Therefore, when establishing an EPT protocol, it is essential to collect enough repetitions to ensure interday stability without inducing a cumulative stimulation effect that might bias the outcomes. The preliminary data suggest that an average of three trials may represent an optimal balance to preserve intraday precision while achieving interday reliability.

From a clinical perspective, the findings of this exploratory study offer practical implications for real-world practice in sports medicine. In clinical environments, where establishing a precise baseline is essential to monitor recovery or evaluate treatment effectiveness prior to return to sport, clinicians carefully weigh measurement precision against time efficiency and patient feasibility. The results in this study suggest that establishing a standardized protocol of three averaged measurements across EST, EMT and EPT, with a between-trial recovery time of 90 s, could serve as a reliable and feasible tool to characterize sensory-motor profiles. Notably, the poorest reliability was observed when relying on a single measurement, indicating that single-trial assessments may lead to inaccuracies in assessment. While data suggest that a three-trial approach represents an optimal balance that maximizes reliability without unnecessarily increasing testing time, it is important to highlight that these reliability values were obtained under strictly controlled conditions. Factors such as consistent electrode placement, recovery intervals, controlled environmental conditions, and a single intra-session examiner must be carefully replicated in clinical settings to ensure threshold stability. Electrical thresholds represent a promising tool to support clinical assessments in active populations. However, these findings remain preliminary, and future research is warranted to validate this protocol and address the existing gaps in clinical populations with sport-related injuries.

An additional consideration for clinical translation relates to the specific demographic profile of the analyzed cohort, which comprised active subjects and predominantly young female participants. Regular exercise is known to induce functional adaptations in the nervous system that support athletic performance [32], while physiological, neurophysiological, and hormonal variations between sexes and ages can significantly influence sensory perception, pain sensitivity, and absolute electrical sensory and motor threshold values [9,33,34]. Within this framework, this protocol suggests that ETT could be used with confidence to establish somatosensory profiles. However, since thresholds were explored in a healthy population, their direct clinical relevance for injured athletes or patients experiencing somatosensory conditions remains hypothetical at this stage. These findings should be utilized as a preliminary methodological guide for active populations, highlighting the need for future clinical trials to investigate the specific behavior of these threshold requirements directly within injured cohorts and more sex- and age-balanced samples.

This exploratory study has several limitations that should be considered when interpreting the findings. First, although previous studies assessing the reliability of electrical thresholds have used comparable sample sizes (*n* = 13) [13], it should be acknowledged that the sample size (*n* = 14) was relatively small and composed mainly of young (mean of 23.57 ± 4.18 years), healthy, active females (71.4% female). Nevertheless, this research was conceived as an exploratory pilot study primarily aimed at establishing preliminary methodological standardization and baseline requirements, a stage where a homogeneous sample is highly useful to minimize confounding biological variability. These features, however, substantially limit the direct generalizability of our findings to broader athletic populations, males, or patients with actual sensorimotor impairments. Therefore, our findings should be interpreted within the context of an initial pilot framework, and future studies should validate these protocols in larger, diverse cohorts and clinical populations. Another limitation concerns the anatomical site of assessment. All measurements were taken exclusively on the dominant forearm. Although this restricts the extrapolation of findings to other body areas, the forearm is widely accepted in QST as a standard location for evaluating somatosensory function in non-pain-referred regions [35], justifying its use in this study. Moreover, assessments were performed by a single examiner, which limited the analysis of inter-rater reliability. The lack of an inter-examiner analysis limits our ability to guarantee the reproducibility of this ETT protocol across different clinicians, an aspect that remains essential for its widespread clinical applicability. Finally, although a rigorous and standardized measurement protocol was implemented to minimize intersubject variability [9], due to technical constraints, it was not possible to quantify the exact distance between the stimulation electrodes and underlying neural structures, which may influence individual threshold values. However, potentially confounding factors were documented—including sex, age, and body mass—which have been reported to influence both sensory and motor thresholds [9,36], thereby enhancing the robustness of our findings. Additionally, it must be acknowledged that other factors known to influence sensory and pain thresholds—such as sleep quality, psychological stress, fatigue, caffeine intake, and hormonal status (e.g., menstrual cycle phases) [37,38]—were not strictly monitored due to the exploratory nature of the study. Although our standardized testing schedule minimized intraday circadian variations, these unmeasured confounding variables should be carefully considered in future research.

## 5. Conclusions

This exploratory study demonstrated that ETT yields good-to-excellent reliability for EST and EPT when multiple trials were averaged, while EMT reliability varied from moderate-to-excellent depending on the performed test. Based on these preliminary findings, implementing a standardized, uniform protocol of three-averaged measurements, with a 90-s recovery interval, appears to offer an optimal and efficient methodological balance to ensure baseline stability across all three thresholds. These results may have direct implications for QST assessment in active populations, helping to standardize protocols and support the use of ETT as a reliable tool for evaluating sensorimotor impairments and monitoring treatment outcomes. However, findings should be interpreted with caution due to methodological limitations. Future research should aim to extend these findings and to evaluate the effectiveness of ETT for detecting sensorimotor changes associated with sport-related injuries and monitoring their longitudinal course.

## Figures and Tables

**Table 1:** Descriptive analysis of the sample.

Outcome	Mean/AF	SD/%
Age (years)	23.57	4.18
Height (m)	1.63	0.12
Body Mass (kg)	69.14	27.64
Sex (women)	10	71.40
Laterality (right)	14	100.00

Categorical variables are expressed as absolute frequencies (AF) and percentages (%) within each group. Quantitative variables are expressed as mean and standard deviation (SD).

**Table 2:** Reliability analysis of the measures for sensory threshold test.

STT		Intraday Reliability					Interday Reliability				
Outcome	*N*	ICC	CI (95%)	*p*	SEM	MDC	ICC	CI (95%)	*p*	SEM	MDC
EST (mA)	1	0.651	(0.206–0.873)	0.004	1.04	2.88	0.783	(0.451–0.925)	<0.001	0.82	2.27
	2	0.817	(0.430–0.941)	0.002	0.71	1.96	0.840	(0.502–0.949)	0.001	0.66	1.83
	3	0.834	(0.483–0.947)	0.001	0.66	1.84	0.852	(0.538–0.952)	0.001	0.63	1.74
	4	0.822	(0.445–0.943)	0.002	0.72	1.99	0.874	(0.609–0.960)	<0.001	0.60	1.67
	5	0.823	(0.448–0.943)	0.002	0.74	2.05	0.884	(0.640–0.963)	<0.001	0.60	1.66

1–5 refer to the number of measures used to obtain the mean. CI (95%): 95% Confidence Interval; EST: Electrical Sensory Threshold; ICC: Intraclass Correlation Coefficient; MDC: Minimal Detectable Change in mA; *N*: number of measurements for the mean; SEM: Standard Error of Measurement in mA; STT: Sensory Threshold Test.

**Table 3:** Reliability analysis of the measures for motor threshold test.

MTT		Intraday Reliability					Interday Reliability				
Outcome	*N*	ICC	CI (95%)	*p*	SEM	MDC	ICC	CI (95%)	*p*	SEM	MDC
EST (mA)	1	0.669	(0.237–0.880)	0.003	1.34	3.71	0.660	(0.221–0.877)	0.004	1.36	3.76
	2	0.791	(0.348–0.933)	0.004	1.09	3.01	0.858	(0.556–0.954)	0.001	0.90	2.48
	3	0.778	(0.308–0.929)	0.005	1.21	3.34	0.855	(0.549–0.954)	0.001	0.97	2.70
	4	0.807	(0.399–0.938)	0.003	1.09	3.02	0.888	(0.653–0.964)	<0.001	0.83	2.30
	5	0.844	(0.513–0.950)	0.001	0.94	2.59	0.895	(0.673–0.966)	<0.001	0.77	2.13
EMT (mA)	1	0.867	(0.638–0.955)	<0.001	1.27	3.52	0.460	(−0.071–0.788)	0.042	2.56	7.08
	2	0.928	(0.774–0.977)	<0.001	0.98	2.71	0.683	(0.012–0.898)	0.024	2.05	5.68
	3	0.940	(0.815–0.981)	<0.001	0.87	2.40	0.699	(0.064–0.904)	0.019	1.94	5.38
	4	0.946	(0.833–0.983)	<0.001	0.79	2.18	0.715	(0.112–0.909)	0.016	1.80	5.00
	5	0.949	(0.841–0.984)	<0.001	0.75	2.09	0.742	(0.197–0.917)	0.010	1.70	4.70

1–5 refer to the number of measures used to obtain the mean. CI (95%): 95% Confidence Interval; EMT: Electrical Motor Threshold; EST: Electrical Sensory Threshold; ICC: Intraclass Correlation Coefficient; MDC: Minimal Detectable Change in mA; MTT: Motor Threshold Test; *N*: number of measurements for the mean; SEM: Standard Error of Measurement in mA.

**Table 4:** Reliability analysis of the measures for pain threshold test.

PTT		Intraday Reliability					Interday Reliability				
Outcome	*N*	ICC	CI (95%)	*p*	SEM	MDC	ICC	CI (95%)	*p*	SEM	MDC
EST (mA)	1	0.842	(0.579–0.946)	<0.001	0.79	2.20	0.819	(0.527–0.938)	<0.001	0.85	2.36
	2	0.916	(0.740–0.973)	<0.001	0.67	1.86	0.963	(0.886–0.988)	<0.001	0.45	1.24
	3	0.942	(0.818–0.981)	<0.001	0.56	1.55	0.945	(0.830–0.982)	<0.001	0.54	1.51
	4	0.944	(0.826–0.982)	<0.001	0.54	1.49	0.960	(0.875–0.987)	<0.001	0.46	1.26
	5	0.952	(0.852–0.985)	<0.001	0.49	1.37	0.964	(0.889–0.989)	<0.001	0.43	1.18
EMT (mA)	1	0.879	(0.665–0.959)	<0.001	1.09	3.03	0.552	(0.054–0.831)	0.016	2.10	5.82
	2	0.941	(0.816–0.981)	<0.001	0.69	1.91	0.803	(0.387–0.937)	0.003	1.26	3.49
	3	0.950	(0.843–0.984)	<0.001	0.62	1.72	0.833	(0.481–0.946)	0.001	1.14	3.15
	4	0.946	(0.831–0.983)	<0.001	0.62	1.71	0.846	(0.521–0.951)	0.001	1.04	2.88
	5	0.958	(0.868–0.986)	<0.001	0.54	1.49	0.866	(0.582–0.957)	<0.001	0.96	2.67
EPT (mA)	1	0.912	(0.749–0.971)	<0.001	1.06	2.93	0.373	(−0.174–0.744)	0.085	2.83	7.83
	2	0.957	(0.866–0.986)	<0.001	0.60	1.67	0.622	(−0.178–0.879)	0.046	1.78	4.94
	3	0.905	(0.703–0.969)	<0.001	0.80	2.22	0.782	(0.321–0.930)	0.005	1.21	3.36
	4	0.835	(0.487–0.947)	0.001	1.01	2.79	0.804	(0.391–0.937)	0.003	1.10	3.04
	5	0.812	(0.415–0.940)	0.002	1.06	2.94	0.853	(0.541–0.953)	0.001	0.94	2.60

1–5 refer to the number of measures used to obtain the mean. CI (95%): 95% Confidence Interval; EMT: Electrical Motor Threshold; EPT: Electrical Pain Threshold; EST: Electrical Sensory Threshold; ICC: Intraclass Correlation Coefficient; MDC: Minimal Detectable Change in mA; *N*: number of measurements for the mean; PTT: Pain Threshold Test; SEM: Standard Error of Measurement in mA.

## Data Availability

The raw data supporting the conclusions of this article will be made available by the authors on request.

## Abbreviations

The following abbreviations are used in this manuscript:

ETT	Electrical Threshold Testing
QST	Quantitative Sensory Testing
EST	Electrical Sensory Threshold
EMT	Electrical Motor Threshold
EPT	Electrical Pain Threshold
ICCs	Intraclass Correlation Coefficients
CEICA	Research Ethics Committee of the Autonomous Community of Aragón
CUSTOS	Data Protection Unit of the University of Zaragoza
STT	Sensory Threshold Test
MTT	Motor Threshold Test
PTT	Pain Threshold Test
